# Sensitivity Analysis of Vagus Nerve Stimulation Parameters on Acute Cardiac Autonomic Responses: Chronotropic, Inotropic and Dromotropic Effects

**DOI:** 10.1371/journal.pone.0163734

**Published:** 2016-09-30

**Authors:** David Ojeda, Virginie Le Rolle, Hector M. Romero-Ugalde, Clément Gallet, Jean-Luc Bonnet, Christine Henry, Alain Bel, Philippe Mabo, Guy Carrault, Alfredo I. Hernández

**Affiliations:** 1 INSERM, U1099, Rennes, France; 2 Université de Rennes 1, LTSI, Rennes, France; 3 Sorin CRM SAS (a LivaNova company), Clamart, France; 4 INSERM, UMR970 Paris Cardio-vascular Research Center, Paris, France; 5 Assistance Publique-Hôpitaux de Paris, Department of Cardiology, Hôpital Européen Georges Pompidou, Paris, France; 6 Paris Descartes University, PRES Paris Sorbonne, Paris, France; 7 CHU Rennes, Department of Cardiology, Rennes, France; 8 INSERM, CIC-IT 1414, Rennes, France; University of California Los Angeles, UNITED STATES

## Abstract

Although the therapeutic effects of Vagus Nerve Stimulation (VNS) have been recognized in pre-clinical and pilot clinical studies, the effect of different stimulation configurations on the cardiovascular response is still an open question, especially in the case of VNS delivered synchronously with cardiac activity. In this paper, we propose a formal mathematical methodology to analyze the acute cardiac response to different VNS configurations, jointly considering the chronotropic, dromotropic and inotropic cardiac effects. A latin hypercube sampling method was chosen to design a uniform experimental plan, composed of 75 different VNS configurations, with different values for the main parameters (current amplitude, number of delivered pulses, pulse width, interpulse period and the delay between the detected cardiac event and VNS onset). These VNS configurations were applied to 6 healthy, anesthetized sheep, while acquiring the associated cardiovascular response. Unobserved VNS configurations were estimated using a Gaussian process regression (GPR) model. In order to quantitatively analyze the effect of each parameter and their combinations on the cardiac response, the Sobol sensitivity method was applied to the obtained GPR model and inter-individual sensitivity markers were estimated using a bootstrap approach. Results highlight the dominant effect of pulse current, pulse width and number of pulses, which explain respectively 49.4%, 19.7% and 6.0% of the mean global cardiovascular variability provoked by VNS. More interestingly, results also quantify the effect of the interactions between VNS parameters. In particular, the interactions between current and pulse width provoke higher cardiac effects than the changes on the number of pulses alone (between 6 and 25% of the variability). Although the sensitivity of individual VNS parameters seems similar for chronotropic, dromotropic and inotropic responses, the interacting effects of VNS parameters provoke significantly different cardiac responses, showing the feasibility of a parameter-based functional selectivity. These results are of primary importance for the optimal, subject-specific definition of VNS parameters for a given therapy and may lead to new closed-loop methods allowing for the optimal adaptation of VNS therapy through time.

## Introduction

Vagus nerve stimulation (VNS) is an approved clinical therapy for medically refractory epilepsy and depression [1–3]. More recently, VNS has been proposed as a promising therapeutic approach for other pathologies, such as heart failure (HF) [4, 5], cardiac arrhythmia [6] or inflammation and auto-immune diseases [7]. One common difficulty in all these clinical applications is to deliver an efficient therapy, while minimizing side effects. This is a particularly complex problem in the case of VNS, since a typical stimulation pattern consists of a set of biphasic pulses, characterized by several parameters (current amplitude, pulse width, number of pulses, interpulse period), delivered through different electrode configurations. Moreover, these VNS patterns may be triggered by the detection of the cardiac activity (synchronous VNS) [8] or applied independently on the cardiac function (asynchronous VNS) [9, 10]. The adaptive, subject-specific, closed-loop definition of VNS parameters seems to be an interesting option [11–13], however, little knowledge is available today on the physiological effects produced by varying VNS parameters in a combined fashion.

Experimental studies have shown that the choice of VNS parameters may have a significant impact on the therapeutic outcome in the context of myocardial ischemia and HF [14]. Also, the acute cardiovascular response to individual VNS parameter variations has been evaluated, concerning the current amplitude [15], the number of pulses [16], pulse width [17], pulse frequency [18–20] or the delay with respect to the detected cardiac cycle, in the case of synchronous VNS [16, 18, 21–23]. More recently, studies have shown that selective acute cardiac responses can be obtained by applying different combinations of VNS parameter values [24, 25]. Moreover, the relative contributions of the direct efferent effect and the centrally-mediated afferent effect of VNS have also been studied [26, 27]. These studies underline the necessity of a time-dependent, subject-specific optimization of VNS parameters and highlight the underlying complexity of such optimization. Nevertheless, these studies are limited by the amount of VNS parameter combinations that are analyzed or the lack of a formal quantitative analysis method of the observed cardiac responses to different VNS configurations. Most of these studies are focused on stimulation applied independently on the cardiac function (asynchronous VNS) and information about cardiovascular response to synchronous VNS parameters is still missing.

In this paper, we propose a formal methodology to analyze the relative significance and the level of interaction between the different VNS parameters, with respect to their effects on acute chronotropic, inotropic and dromotropic responses, in the case of synchronous VNS. An original experimental design based on an optimized Latin Hypercube sampling (LHS) method has been proposed, to generate a set of 75 different VNS parameter configurations to be used in experimental evaluation. These LHS-based VNS configurations were applied to anesthetized healthy sheep, while acquiring the most significant cardiovascular variables. Subject-specific estimations of the cardiac responses to unobserved VNS parameter values are obtained through a surrogate model, fitting the observed data acquired from each sheep. These surrogate models were used as input to a Sobol sensitivity analysis method, to derive quantitative markers of the significance of each VNS parameter and their combinations on each cardiac effect. Results obtained from 6 sheep, are presented and their impact on the design of subject-specific, closed-loop VNS therapy is discussed.

## Methods

### Experimental protocol

Data for this study were obtained from six healthy sheep (6.8 ± 1.9 months old, 37.8 ± 3.6 kg), following a protocol approved by the French local ethics committee for animal experimentation (“Comité d’thique en matière d’expérimentation animale”, Paris Descartes, Paris, France). Sheep were initially (induction) anesthetized by propofol (4mg/kg/min) and surgery was performed under isoflurane (1.5%). The intra-cardiac electrogram (EGM) is measured via the SonRTip^™^ lead (Sorin Group Italia, Saluggia, Italy) implanted into the right ventricle. Pressure and electrical probe sensor (Millar Instruments Inc., Houston, USA) was implanted into the left ventricle. A bipolar stimulation cuff (“C4D3-1”, Obelia or “EquiCurl”, Sorin Group Italia, Saluggia, Italy) was placed on the right vagus nerve, at a cervical site. The aortic artery dissection is realized at the cervical level, at an equal distance space between the head and the trunk. The vagus nerve (VN) is gently removed from the aortic sheath and the cuff is placed around the VN.

After a verification stage of the implanted instrumentation (in particular VNS electrode impedance and EGM quality), the experimental evaluation of the proposed control system was performed using Etomidate (100 mcg/kg/min) as anesthetic agent. The surface ECG, the EGM, the left intra-ventricular pressure, and the body temperature were monitored and recorded during the whole procedure. Breathing was artificially controlled at 0.3 Hz (18 breaths/min).

A programmable external neurostimulator (Prototype INTENSE v1.2, Sorin CRM SAS, Clamart, France), driven by a custom real-time control application allowing for the definition of complex temporal stimulation waveforms and dynamic programming of stimulation parameters, was used to apply VNS to the animals [13]. This neurostimulator provides VNS in constant-current mode, through the use of a set of distributed stimulation units. More details on the electronic architecture of the stimulation instrumentation are provided elsewhere [28].

### Vagus nerve stimulation parameters

**Fig 1:**
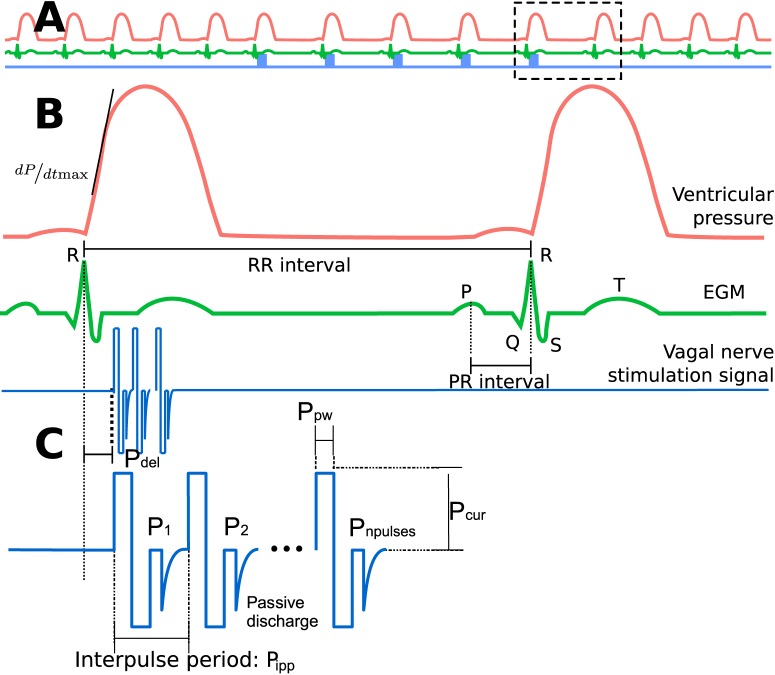
Diagram depicting the main signals and the VNS parameters analyzed in this paper. A) Representation of the left ventricular pressure signal (red), electrogram (EGM, green) and Vagus Nerve Stimulation signal (blue). B) Zoom displaying the main markers extracted from each beat: the inter-beat interval (RR interval) representing the chronotropic effect, the interval between the P-wave and the R-wave (PR inteval) used as a marker of the dromotropic effect and the maximum of the first of the *P*_*lv*_ signal (dPdtmax). C) A typical VNS burst delivered synchronously with a cardiac beat (after a given delay *P*_del_), showing the VNS parameters studied in this paper.

Fig 1C represents a typical stimulation burst applied to the vagus nerve in a synchronous approach. Each burst is characterized by a set of five parameters, that can be represented in a vector denoted

$$\begin{array}{c} S=[P_{\text{cur}},P_{\text{npulses}},P_{\text{pw}},P_{\text{ipp}},P_{\text{del}}], \end{array}$$ (1)

including the delivered current amplitude (*P*_cur_, mA), the number of delivered pulses (*P*_npulses_), the pulse width (*P*_pw_, ms), the interpulse period (*P*_ipp_, ms) and the delay between the detected cardiac event and VNS onset (*P*_del_, ms).

In this paper, a given realization (realization *r*) of vector *S*, having particular values for each parameter, will be noted *S*_*r*_. The notation *S*_*r*_[*i*] represents the value of the *i*th element of vector *S*_*r*_.

The parameter space studied in this work is thus a five-dimensional space, delimited within the support defined by the ranges in Table 1. These ranges were defined according to our previous experience with a similar experimentation setup [24], and the objective of keeping moderate bradycardia effects, with instantaneous RR intervals below 1100ms. During the application of each VNS configuration *S*_*r*_, sheep were continuously monitored to detect strong bradycardia and adverse events. If a strong bradycardia is provoked by *S*_*r*_, (RR interval higher than 1100ms), cardiac pacing is automatically activated with an interbeat interval of 1100ms. Also, side effects (coughs or laryngeal muscle activation) were identified by visual inspection. Configurations *S*_*r*_ provoking strong bradycardia or adverse events were removed from the analysis.

**Table 1:** Vagus nerve stimulation parameter ranges.

Parameter	Notation	Range
Current	*P*_cur_	0.2 mA to 1 mA
Number of pulses	*P*_npulses_	1 to 4
Pulse width	*P*_pw_	0.05 ms to 0.20 ms
Interpulse period	*P*_ipp_	24.4 ms to 47 ms
Delay	*P*_del_	16 ms to 156 ms

Obviously, it is unfeasible to analyze this five-dimensional space using a full factorial design, since it would lead to a very high number of *S*_*r*_ to be experimentally tested. The following section presents the experimental design chosen in this work, to optimize the definition of the configurations *S*_*r*_ that will be applied to the animals.

### Experimental design

In order to improve the exploration of the parameter space of *S*, a space-filling design based on an optimized Latin Hypercube Sampling (LHS) method [29] was used to generate a set $S_{e}=\left\{S_{1},...,S_{N}\right\}$ of *N* = 75 random VNS parameter configurations inside a five-dimensional unit hypercube. This method ensures that the space is explored evenly, while maximizing the mean distance from each point to all other points in the experimental design. Then, each point was rescaled to the practical values of parameter ranges in Table 1. In the particular case of *P*_npulses_, the rescaled value was rounded to the nearest integer. The experimental design generated with the LHS method, $S_{e}$, was generated only once. All 6 sheep in the protocol were stimulated with the same set of VNS configurations, applied in a different random order. Each VNS configuration used for animal experimentation $S_{n}\in S_{e}$ was applied during a 30 s stimulation period (${\text{VNS}}_{\text{n}}^{\text{on}}$) and was preceded by a resting, non stimulation period of 40 s (${\text{VNS}}_{\text{n}}^{\text{off}}$).

### Data processing

In total, 75 VNS configurations were applied to 6 sheep, providing 450 signal segments. Each segment is constituted of a 40 s ${\text{VNS}}_{\text{n}}^{\text{off}}$ period, immediately followed by a 30 s ${\text{VNS}}_{\text{n}}^{\text{on}}$ period. These segments were automatically processed offline and manually verified.

The chronotropic markers were extracted from EGM signals (see Fig 1B). R-waves were detected with a method previously proposed and evaluated by our team [30] and the obtained beat-to-beat RR intervals were used as marker of the chronotropic modulation. P-waves were detected in a second phase using the surface ECG, by applying a model-based research method within a time support of 120 ms preceding each detected R-wave [31]. The beat-to-beat PR intervals were used for studying the dromotropic response. The marker of the inotropic effect was extracted from the intraventricular pressure signal, by measuring the maximum rate of change (dPdtmax) of this signal for each cardiac cycle. Finally, a quantification of the cardiac effect provoked by each *S*_*n*_ was defined as the relative change of each cardiac marker with respect to the baseline value, observed during ${\text{VNS}}_{\text{n}}^{\text{off}}$ period:

yc(Sn)=ΔcSn100=cVNSnonSn-cVNSnoffSncVNSnoffSn;c∈{RR,PR,dPdtmax}, (2)

where $c_{{\text{VNS}}_{\text{n}}^{\text{off}}}^{S_{n}}$ is the average cardiac marker value of the last ten beats in ${\text{VNS}}_{\text{n}}^{\text{off}}$ and $c_{{\text{VNS}}_{\text{n}}^{\text{on}}}^{S_{n}}$ is the median value of the corresponding parameter calculated between beats 4 and 23 of the ${\text{VNS}}_{\text{n}}^{\text{on}}$ period. The first three beats were excluded in order to minimize the effect of the transient response to the stimulation.

### Surrogate model

The analyses and methods used in this work require an estimate of the cardiac effect produced by any VNS parameter configuration *S*_*m*_, within the ranges shown in Table 1. Due to the finite experimental time and as exposed previously, we dispose of *N* = 75 observations *y*_*c*_(*S*_*n*_) for each cardiac marker *c*, within the five-dimensional space of *S*. It is thus necessary to estimate all values ${\hat{y}}_{c}\left(S_{m}\right)$ as a function of the observed data *y*_*c*_(*S*_*n*_), through a surrogate model. In this work, we apply a Gaussian process regression (GPR) model, by defining an estimator of the form:

$$\begin{array}{c} {\hat{y}}_{c}\left(S_{m}\right)={\text{B}}_{\text{c}}S_{m}+Z_{c}\left(S_{m}\right). \end{array}$$ (3)

where **B**_**c**_ *S*_*m*_ is typically a linear regression model and *Z*_*c*_(*S*_*m*_) is a Gaussian process with zero mean and covariance function *C*_*θ*_, which depends on hyperparameters *θ*. A detailed presentation of this family of interpolators can be found in [32, 33].

In this work, since no *a priori* knowledge is known on the function mapping *S* to a given cardiac effect, the most simple regression model has been used, assuming that **B**_*c*_ *S*_*m*_ = *μ*_*c*_, where *μ*_*c*_ is the mean effect of the observed data on cardiac effect *c*. Furthermore, it is assumed that *Z*_*c*_(*S*_*m*_) is a stationary Gaussian process described by a covariance function *C*_*c*,*θ*_(*S*_*n*_, *S*_*m*_) = *K*_*c*,*θ*_(‖*S*_*n*_ − *S*_*m*_‖) that depends on the unknown hyperparameters *θ*, that should be identified, and on the distance between the points in the input space. Three well-known functions were evaluated for *K*_*c*,*θ*_: exponential, Gaussian or the Matérn family of covariance functions [33]. The best fit on the data, for all GPR models, was obtained using the Matérn 5/2 covariance function, which was kept for all developments in this work. Therefore, in order to obtain each estimator ${\hat{y}}_{c}\left(S_{m}\right)$, a training phase is applied to estimate the *θ* parameters of the Matérn 5/2 covariance function from a subset of the observed data, using a maximum likelihood loss function. Once this training phase is finished, each new unobserved ${\hat{y}}_{c}\left(S_{m}\right)$ can be estimated using as only input the distances of *S*_*m*_ to the experimental design $S_{e}$.

For each sheep, three GPR models were created to estimate each cardiac effect (ΔRR, ΔPR and ΔdPdtmax) using DiceKriging R package [34]. In order to evaluate the accuracy of each model, the observed VNS sequences of each individual were randomly partitioned in a training group and testing group in a 3:1 proportion. This random partition was repeated 1000 times in a cross-validation approach, using as performance criteria the log-likelihood function observed in the test group. The model with the highest log-likelihood was selected and used for the sensitivity analyses described in the next section. To ease the presentation of the surrogate model performances, the root mean squared error (RMSE) associated with the selected model was calculated.

### Global sensitivity analysis

Once the surrogate model is defined to estimate each cardiac effect for any value of VNS parameters, a global sensitivity analysis method can be applied to quantitatively characterize the relative significance of each VNS parameter on each cardiac effect. In this work, we applied a global, variance-based sensitivity analysis method: Sobol’s variance decomposition.

In Sobol’s method, the effect of a parameter is measured relative to the variability of the observed output (*Var*[*y*_*c*_]). Assuming that the parameters are statistically independent, an ANOVA decomposition can separate the fraction of *Var*[*y*_*c*_] attributed to the variability of each *S*[*i*]:

$$\begin{array}{c} Var\left[y_{c}\right]=\sum_{i}Var\left[y_{c}|S\left[i\right]\right]+\sum_{i}\sum_{j>i}Var\left[y_{c}|S\left[i\right],S\left[j\right]\right]+\cdots \end{array}$$ (4)

Since the solution to multivariate conditional variances are difficult to calculate, a Monte-Carlo approach is the most commonly used approach, requiring a significant amount of evaluations. In this work, Sobol’s indices were calculated using Monte-Carlo evaluations of 10^6^ random *S*_*m*_, defined within the ranges shown in Table 1. More details on this algorithm are available in [35, 36].

Eq (4) can be divided by *Var*[*y*_*c*_] so that each effect is represented within the support [0, …, 1]. Moreover, each term of Eq (4) can be interpreted in a particular manner:

- A *main effect*, *first-order effect* or *non-interacting effect*, denoted *E*_*i*_ = *Var*[*y*_*c*_|*S*[*i*]], represents the effect on the output explained only by the variability of parameter *S*[*i*], while leaving all other parameters constant.
- An *interactive effect*, or *nth-order effect* (*n* > 2), denoted *E*_*i*…*k*_ = *Var*[*y*_*c*_|*S*[*i*], …, *S*[*k*]], is the effect explained by the common variability of several parameters *S*[*i*], …, *S*[*k*].
- A *total effect*, defined as *E*_*Ti*_ = *E*_*i*_ + ∑_*j*_ *E*_*ij*_ + ∑_*j*,*m*_ *S*_*ijm*_ + …, characterizes the effect provoked by *S*[*i*] (or the main effect, *E*_*i*_), plus the effect produced by all possible interactions with the other parameters.

In this paper, we are interested in measuring the main and total effects of each *S*[*i*]. Moreover, we also calculate the second and third order effects, to explore the part of the cardiac responses due to parameter interactions. The sensitivity R package was used for calculations [37]. Finally, in order to estimate robust inter-individual Sobol sensitivity markers, a bootstrap method has been applied. *E*_*i*_ and *E*_*Ti*_ markers obtained from the Monte-Carlo evaluation for each sheep were used as input to a bootstrap method based on random sampling with replacement (using the “boot” R package). *R* = 10000 realizations were obtained in order to estimate the mean and standard deviation of inter-individual *E*_*i*_ and *E*_*Ti*_ values (calculated for all sheep), as well as their corresponding 95% (percentile) confidence intervals, for each sensitivity marker and for all cardiac effects.

## Results

### Cardiac autonomic responses to different VNS parameters

**Fig 2:**
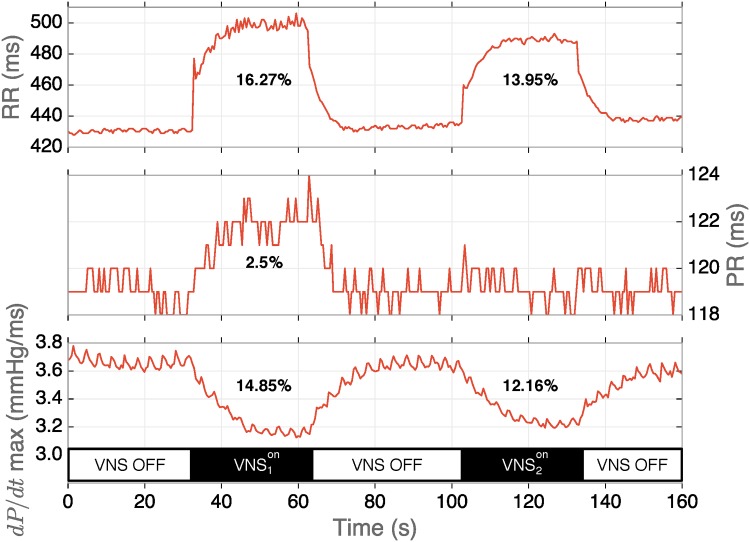
Example of acute cardiac effects provoked by two different sets of VNS parameters. Sequence ${\text{VNS}}_{\text{1}}^{\text{on}}$ was applied with parameters *S*_1_ = [0.8 mA, 2 pulses, 0.05 ms, 21.3 Hz, 125 ms], while sequence ${\text{VNS}}_{\text{2}}^{\text{on}}$ with *S*_2_ = [0.4 mA, 2 pulses, 0.2 ms, 21.3 Hz, 125 ms].

An example of the application of two VNS configurations and their corresponding chronotropic, dromotropic and inotropic markers is depicted in Fig 2. In this example, ${\text{VNS}}_{\text{1}}^{\text{on}}$ was applied with parameters *S*_1_ = [0.8 mA, 2 pulses, 0.05 ms, 21.3 Hz, 125 ms] and ${\text{VNS}}_{\text{2}}^{\text{on}}$ with *S*_2_ = [0.4 mA, 2 pulses, 0.2 ms, 21.3 Hz, 125 ms]. It can be observed that ${\text{VNS}}_{\text{1}}^{\text{on}}$ provoked a higher chronotropic, dromotropic and inotropic responses than ${\text{VNS}}_{\text{2}}^{\text{on}}$. Interestingly, ${\text{VNS}}_{\text{2}}^{\text{on}}$ shows slightly reduced chronotropic and inotropic effects (respectively 13.95% and 12.16%) with respect to ${\text{VNS}}_{\text{1}}^{\text{on}}$ (respectively 16.27% and 14.85%), but with no dromotropic response, whereas a negative effect could be expected [38]. This example shows how VNS parameter interactions may provoke functionally selective responses and underlines the complexity of the optimal definition of VNS parameters.

### Surrogate model

After manual verification, some sequences were removed due to signal noise, yielding incorrect detection of cardiac effects. Also, as stated above, sequences corresponding to VNS configurations leading to extreme bradycardia or adverse events were removed. The second column of Table 2 shows the total number of sequences available for each sheep.

**Table 2:** RMSE of the best surrogate model for each cardiac effect and each sheep.

Subject	Number of sequences	RMSE		
		${\hat{y}}_{\Delta \text{RR}}$	${\hat{y}}_{\Delta \text{PR}}$	y^dPdtmax
		(ms)	(ms)	(mmHg/ms)
Sheep 1	69	1.56	3.78	1.92
Sheep 2	75	1.59	2.22	2.39
Sheep 3	75	2.75	1.40	1.77
Sheep 4	71	5.01	1.24	3.54
Sheep 5	68	2.73	3.06	2.13
Sheep 6	75	1.93	1.01	1.31

Table 2 also shows the accuracy of the best GPR model obtained for each sheep, for each cardiac effect in terms of RMSE. In all cases, a suitable model could be found, with RMSE lower than 5 ms for chronotropic and dromotropic effects, and 4 mmHg/ms for the inotropic effect. RMSE are slightly higher concerning chronotropic and intropic models for sheep 4 and for the dromotropic model for sheep 1.

**Fig 3:**
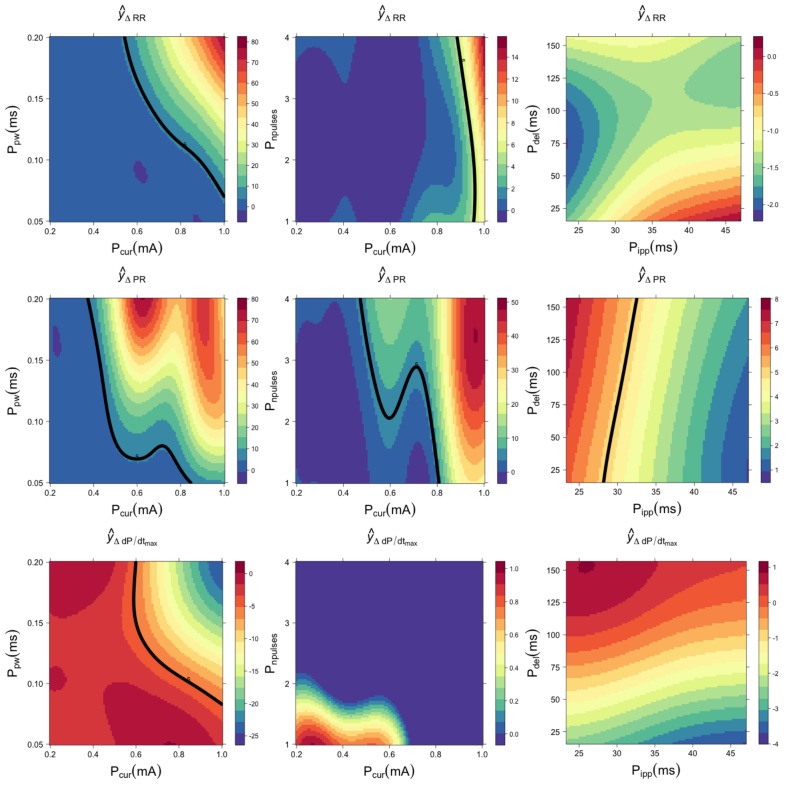
Cardiac responses to different combinations of VNS parameters, estimated with a Gaussian process regression for sheep 1. The chronotropic (${\hat{y}}_{\Delta \text{RR}}$), dromotropic (${\hat{y}}_{\Delta \text{PR}}$) and inotropic (y^dPdtmax) responses are shown in the first, second and third rows, respectively. Note the different scales of each colormap. The continuous black line represents a reference threshold of 5% variation with respect to the baseline value.

Using the GPR model, it is possible to construct surface response graphs, allowing us to visualize the cardiac effect provoked by simultaneously varying a pair of VNS parameters, while leaving the other parameters in constant values. Fig 3 shows illustrative surfaces generated by chronotropic, dromotropic and inotropic GPR models for sheep 1. In this example, surfaces on the left column were generated by varying *P*_pw_ and *P*_cur_, and fixing *P*_npulses_ = 4 pulses, *P*_ipp_ = 31.25 ms and *P*_del_ = 94 ms. Surfaces on the center column were obtained by fixing *P*_pw_ = 0.1 ms and varying *P*_npulses_ and *P*_cur_. Surfaces on the right column were obtained with *P*_cur_ = 0.6 mA while modifying *P*_del_ and *P*_ipp_. Although we are interested in the continuous modification of the observed cardiac effects, represented by the colormap on these surfaces, an effect threshold of 5% with respect to the baseline value is also depicted (continuous black line), as a reference. It is important to note that all the parameter sensitivity calculations presented in the following sections were performed on these continuous values and not on a binary (on-off) effect, as proposed in other works.

The illustrative surfaces presented in Fig 3 show the complexity of the autonomic responses to stimulation parameters. For all cardiac responses, pulse current, pulse width and number of pulses have dominant effects over interpulse period and delay, which appear as negligible parameters. The surface responses, generated by varying *P*_npulses_ vs *P*_cur_ and *P*_del_ vs *P*_ipp_, are weaker in magnitude compared to those produced with *P*_pw_ and *P*_cur_. The surfaces profiles also illustrate the difficulty of using predefined VNS parameter values. In fact, similar effects can be produced by several sets of parameters (see for example the dromotropic response to *P*_pw_ and *P*_cur_). These results also show that the bradycardia threshold of 5%, frequently used in the literature, highly depends on specific combinations of stimulation parameters, and not only on a single parameter. Although the general response of the cardiac effect was similar for all sheep, these surfaces presented differences between sheep, showing the necessity of a subject-specific tuning of stimulation parameters. Surface responses for all other sheep, using the same VNS parameter values, are shown in S1–S5 Figs.

Another important point illustrated by these surfaces is the functional selectivity associated with each cardiac effect. For instance, in Fig 3 upper-left surface, it can be observed that a *P*_pw_ = 0.2 and *P*_cur_ = 1mA provoke approximately an 80% increase in RR interval with respect to the baseline value. With the same *P*_cur_ = 1mA value and reducing *P*_pw_ to about 0.1, it is possible to observe a significant drop in the chronotropic response (passing from 80% to 20%), while keeping a dromotropic effect higher than 45% above the baseline value (seen in the same part of the plane, for the surface in the middle-left panel). The same reasoning can be applied to all other surfaces in Fig 3. This is especially obvious on the *P*_npulses_/*P*_cur_ plane, where significant chronotropic effects can be observed while the corresponding inotropic responses are negligible, particularly for high values of current and number of pulses. This selectivity concerning chronotropic and inotropic effects is highly reproducible for all sheep in the selected *P*_npulses_/*P*_cur_ plane (see S1–S5 Figs). The mean chronotropic effect in this plane for the 6 sheep is equal to 58% of the maximum response, while the inotropic effect is equal to 1.9%. The difference between these autonomic effects was found statistically significant with a Wilcoxon paired test (p-value < 0.001). This functional selectivity is particularly important for the setting of stimulation parameters, while seeking a set a parameters that provoke significant chronotropic responses, associated with an efficient vagal stimulation, while preserving cardiac contractility.

### Sobol sensitivity analysis

**Fig 4:**
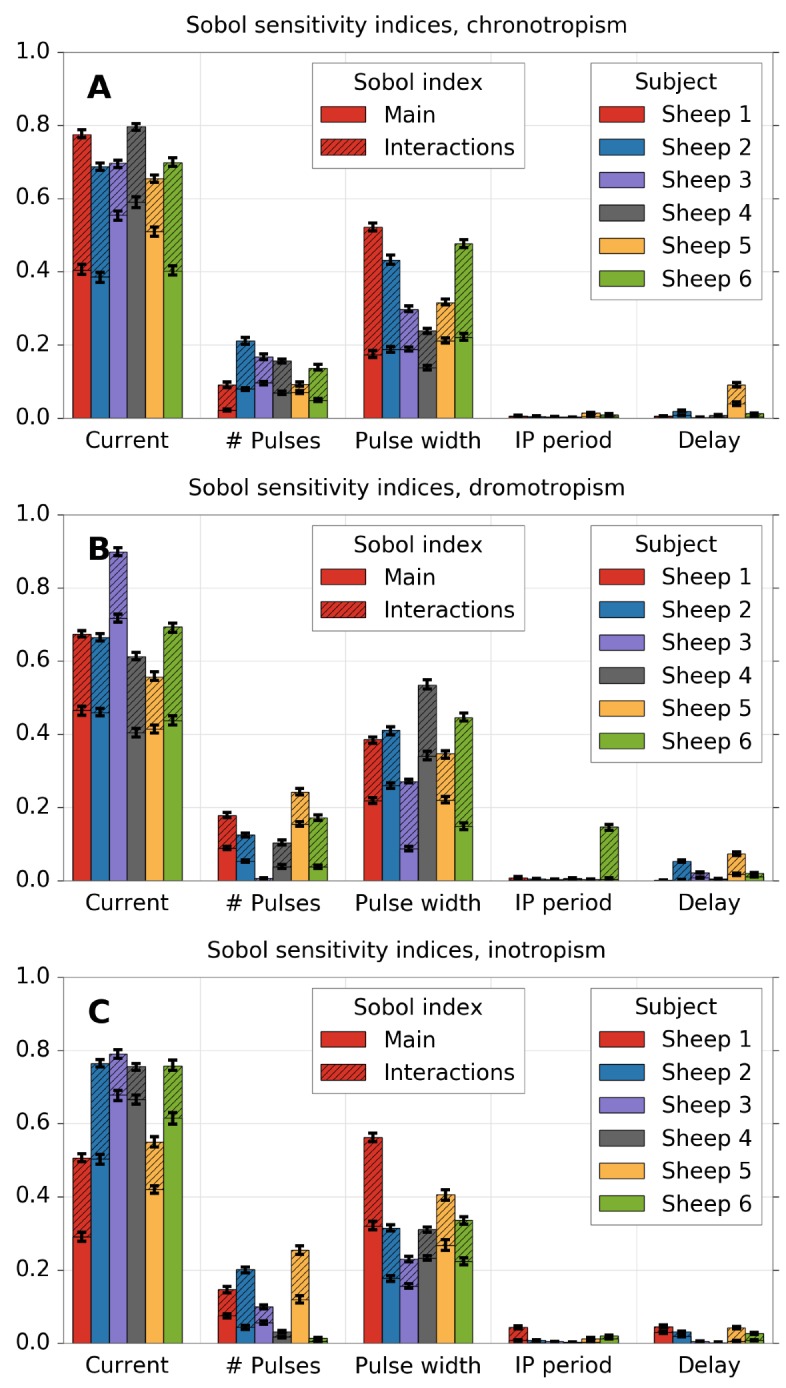
Estimation of Sobol’s main and interactions indices for each VNS parameter on each cardiac effect. A) chronotropic effect, B) dromotropic effect and C) inotropic effect. Total effects are equal to the sum of main and interactions effects (the whole color bar).

Fig 4 shows the estimated main and total Sobol sensitivity indices for the chronotropic (panel A), dromotropic (panel B) and inotropic (panel C) effects, for all sheep. Concerning the main effect (solid bars), a relative significance of each VNS parameter can be established for all cardiac responses. It can be also observed a significant inter-individual variability. This variability depends on the VNS parameter, and is more pronounced for the main effect than for the total effect. More importantly, it can be seen that the differences between main and total effects are high, showing the importance of interacting effects concerning the autonomic responses to different combinations of VNS parameters.

For chronotropism (A), only current, pulse width and number of pulses present a significant total effect. This result suggests that all the interactions can be attributed to the six combinations of second-order effects among these parameters or to the third-order effect of them. The same pattern can be observed for dromotropism (B) and inotropism (C). In all cases, since the main effect of *P*_ipp_ and *P*_del_ is close to zero, the source of the total effect in these cases is mainly provoked by interactive effects with current, pulse width or number of pulses.

**Fig 5:**
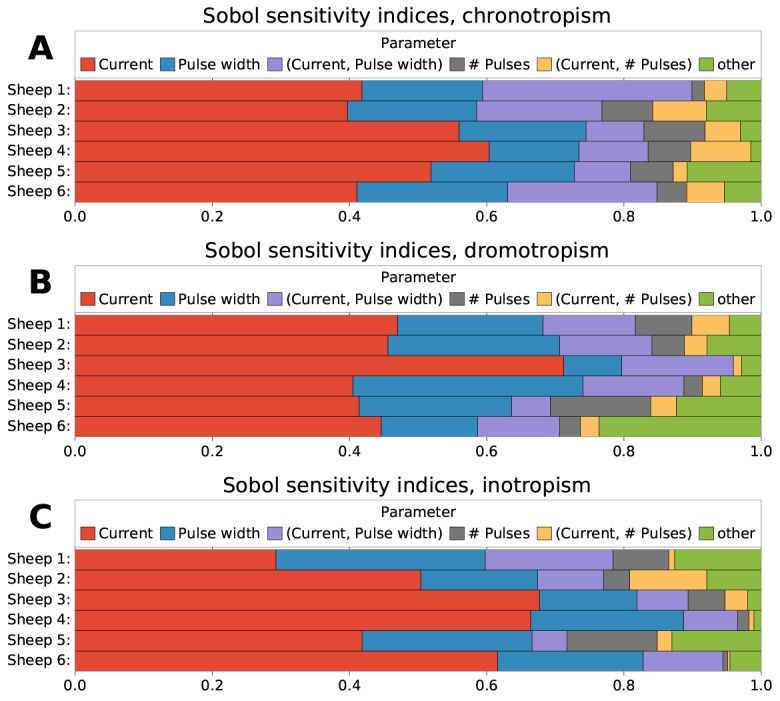
Relative sensitivity of each VNS parameter and VNS parameter combination, according to Sobol’s first order and interaction indices. A) chronotropic effect, B) dromotropic effect and C) inotropic effect. Only the five largest indices are shown, while the rest are summed together (“other”).

Fig 5 shows the results of the same kind of analysis using Sobol indices, but using first, second and third order indices. Since the number of effects increases exponentially, only the five larger effects are shown. The other effects are summed and labelled as “other”. The identification of the exact interactive effects that contribute to the output variability provide novel results that were impossible to calculate in previous works.

For chronotropism (Fig 5A), at least 90% of the variability can be explained from the three most important VNS parameters and their interactions. Among these, the interaction of current and pulse width can explain around 8–20%; the third most important effect. The interaction of current and the number of pulses is also important, but not for all individuals. Concerning dromotropism (Fig 5B), at least 80% of the variability is accounted by almost the same parameters. A detailed verification showed that these interactions come from all the second-order effects associated with delay and interpulse period, respectively. However, none of these interactions was large enough by itself to be classified among the top five effects. Finally, concerning inotropism (Fig 5C), results also show a significant effect from the interaction of current and pulse width.

Table 3 presents the mean and standard deviations of main and total indices over all sheep, for each parameter and each cardiac effect, as well as the confidence intervals obtained through the bootstrap method. The total interactive effects were calculated as the difference between main and total indices. The pulse current amplitude is the most influential parameter, for all cardiac effects, with the main sensitivity indices comprised between 47.45% and 52.82%. The second parameter in terms of significance is pulse width, explaining between 18.60% and 22.94% of the variability. In third place, the number of pulses can account for less than 7% of the variability, while the interpulse period and delay have only negligible contributions.

**Table 3:** Sensitivity analysis results for chronotropic dromotropic and inotropic responses. Main, total and interaction effects are expressed as percentage, along with their corresponding 95% confidence intervals [c.i. 95%], computed using a bootstrap approach.

Chronotropic	Main (%)	Total (%)	Interactions (%)
***P***_**cur**_	47.45 (± 8.83)	71.79 (± 5.52)	24.34 (± 9.35)
	[41.50, 54.01]	[68.11, 76.15]	[17.89, 30.93]
***P***_**npulses**_	6.37 (± 2.58)	14.23 (± 4.62)	7.86 (± 3.56)
	[4.16, 7.97]	[11.01, 17.68]	[4.98, 10.41]
***P***_**pw**_	18.60 (± 2.97)	38.01 (± 11.21)	19.42 (± 10.36)
	[15.96, 20.41]	[29.79, 46.12]	[12.85, 27.58]
***P***_**ipp**_	0.21 (± 0.16)	0.64 (± 0.41)	0.43 (± 0.25)
	[0.10, 0.34]	[0.38, 0.99]	[0.27, 0.64]
***P***_**del**_	0.88 (± 1.47)	2.26 (± 3.36)	1.34 (± 1.9)
	[0.22, 2.68]	[0.70, 6.42]	[0.48, 3.69]
**Dromotropic**	Main (%)	Total (%)	Interactions (%)
***P***_**cur**_	48.26 (± 11.71)	68.31 (± 11.67)	20.05 (± 3.75)
	[42.74, 62.44]	[61.97, 79.62]	[17.08, 22.53]
***P***_**npulses**_	6.16 (± 5.34)	13.71 (± 8.00)	7.55 (± 4.17)
	[3.01, 10.94]	[6.95, 18.70]	[4.03, 10.17]
***P***_**pw**_	21.21 (± 8.78)	39.87 (± 8.96)	18.66 (± 5.93)
	[14.75, 27.44]	[33.39, 46.43]	[15.49, 24.70]
***P***_**ipp**_	0.13 (± 0.09)	2.85 (± 5.74)	2.71 (± 5.6)
	[0.04, 0.19]	[0.44, 9.91]	[0.35, 9.72]
***P***_**del**_	0.61 (± 0.67)	2.86 (± 2.83)	2.24 (± 2.51)
	[0.19, 1.17]	[1.11, 5.26]	[0.61, 4.53]
**Inotropic**	Main (%)	Total (%)	Interactions (%)
***P***_**cur**_	52.82 ± 15.36	68.70 ± 12.45	15.87 ± 6.61
	[39.60, 62.34]	[56.97, 76.55]	[11.66, 21.43]
***P***_**npulses**_	5.19 ± 4.13	12.43 ± 9.48	7.23 ± 6.25
	[2.53, 8.57]	[5.57, 19.29]	[3.12, 12.10]
***P***_**pw**_	22.94 ± 5.93	35.92 ± 11.40	12.97 ± 6.18
	[18.93, 27.65]	[29.21, 46.83]	[9.55, 19.02]
***P***_**ipp**_	0.37 ± 0.46	1.48 ± 1.52	1.11 ± 1.26
	[0.10, 0.83]	[0.676, 3.120]	[0.49, 2.63]
***P***_**del**_	0.96 ± 1.13	2.51 ± 1.84	1.54 ± 1.29
	[0.28, 2.01]	[1.05, 3.71]	[0.76, 2.71]

Wilcoxon paired tests were performed to measure statistical differences between cardiac effects. Significant differences (p-value<0.05) were found between chronotropic and inotropic responses for the main effects associated with *P*_cur_ and interactions effects associated with *P*_cur_, *P*_pw_ and *P*_npulses_. These results suggest that functional selectivity cannot be obtained by varying only one VNS parameter. However, efficient functional selectivity between chronotropic and inotropic responses may be produced by jointly modulating *P*_cur_, *P*_pw_ and *P*_npulses_.

## Discussion

The objective of this study was to investigate, using formal experimental and analysis methods, the influence of VNS parameters on the acute autonomic chronotropic, dromotropic and inotropic responses. The quantitative analysis of the acute cardiac effects provoked by the interaction of VNS parameters provides novel results that were impossible to calculate in previous works. The experimental protocol was designed using LHS in order to ensure a uniform distribution of the VNS configurations applied experimentally. A surrogate model method, based on Gaussian process regressions, was applied to estimate the effects of VNS for unobserved sets of parameters. Global sensitivity analyses were performed on the obtained models in order to quantitatively characterize the influence of each VNS parameter and their interactions on the three observed cardiovascular responses.

This original approach allowed us to quantify the influence of VNS parameters on the main acute cardiac effects of VNS, while keeping a reasonable number of experimental sequences. To our knowledge, such methodology has never been applied to this field. Indeed, most published works only consider the individual sensitivity of one parameter [15, 18] on a limited number of physiological variables. The work of Kong et al. [14] studied the effect of a combination of VNS parameters, and proposed an interesting experimental approach based on the uniform design method. However, the number of VNS parameter combinations used experimentally remained too low to be useful for a formal quantitative analysis of global parameter sensitivity.

Another original aspect of this work is the joint analysis of the three most significant acute cardiac autonomic effects (chronotropic, dromotropic and inotropic responses), studied synchronously with the same set of VNS parameters. Although the inter-individual variability was high, a clear relative significance of VNS parameters appears from the results. The VNS parameters provoking the greater effect were, in decreasing order, *P*_cur_, *P*_pw_, the combination of *P*_cur_ and *P*_pw_, and *P*_npulses_. The effects of *P*_ipp_ and *P*_del_ seems to be lower. The significant sensitivity of the three cardiac effects to *P*_cur_ was expected, as already known from previous studies [15, 25, 39]. However, our work shows that around one third of the total sensitivity to *P*_cur_ is due to interacting effects with other VNS parameters. Our sensitivity analysis also highlights the importance of *P*_pw_ on the three studied cardiac effects. Studies dealing with the impact of pulse width on cardiac effects offer contradictory conclusions [17, 39]. These discrepancies may be explained by the significant interactions between *P*_pw_, *P*_cur_ and *P*_npulses_. Our study clearly establishes the sensitivity of the acute cardiac responses to changes in pulse width, concerning individual and interacting effects, in the case of synchronous pulse trains. Concerning *P*_npulses_, results show that it provokes significant effects, even if the influence is low compared to *P*_cur_, as reported in [15]. This work further shows that the sensitivity of *P*_npulses_ is, in most of the cases, lower than that provoked by the interactions between *P*_cur_ and *P*_pw_.

Numerous studies in the literature concerning synchronous VNS have pointed out a significant and non-linear effect of *P*_del_ on cardiac responses [16, 18, 21–23]. The low sensitivity to *P*_del_ in our study is mainly related to the range defined in our experiments (16 to 156 ms). The delay values were limited here to 156 ms in order to avoid a cross-talk phenomenon of our current prototype between the two stimulation leads (the cuff used for VNS and the intracardiac lead used for cardiac pacing) that may provoke cardiac arrhythmia.

To our knowledge, this is the first work evaluating the influence of interpulse period, *P*_ipp_, which showed negligible main sensitivity effects in this analysis. It should be recalled here that all the results in this work should be considered only within the domain of cardiac-synchronized VNS, and that an equivalence with asynchronous VNS is difficult to obtain. This is particularly true for the *P*_ipp_ parameter, which should not be confused with the inverse of the pulse frequency parameter used in asynchronous VNS. For instance, a sheep with an average heart rate of 120 beats per minute would show similar cardiac responses when stimulated with a synchronous VNS pattern using *P*_npulses_ = 1 and with an asynchronous VNS with a pulse frequency of 2Hz (all other common parameters being equal). Obviously, the response to synchronous stimulation is even more difficult to compare to asynchronous VNS when taking into account natural heart rate variability and the cases in which *P*_npulses_ > 1, with the effect of specific parameters *P*_del_ and *P*_ipp_. Moreover, results show that the cardiac effect produced by modifications in *P*_ipp_ highly depends on other parameters, suggesting that the sensitivity to common parameters between synchronous and asynchronous VNS (*P*_cur_, *P*_pw_), may also be significantly different. Therefore, our results on *P*_ipp_ cannot be easily compared to previous works focused on the effect of pulse frequency, studied in asynchronous, continuous VNS with values usually below 10Hz [17–20].

Results from the GPR modeling phase and from the global sensitivity analysis also suggest that functional selectivity can be hardly provoked by modifying only one VNS parameter. However, as it was shown in Fig 3 and Table 3, this functional selectivity can be obtained by modifying a combination of VNS parameters. This can be observed by the difference between the total and the main effects provoked by each VNS parameter, for all cardiac responses. For instance, interaction effects for *P*_cur_, *P*_pw_ and *P*_npulses_ are significantly different between chronotropic and inotropic effects. The interaction between *P*_cur_ and *P*_npulses_ seems particularly important in this functional selectivity. Functional selectivity, induced by varying jointly these stimulation parameters, is characterized by a consistent negative chronotropic response associated with a preserved cardiac contractility.

Finally, these results show the complexity of the optimization of VNS therapy during the titration phase, due to significant inter and intra-patient variability of the three studied cardiac effects. These facts underline the necessity of an automated titration, integrating closed-loop control methods, in order to optimize the response to the therapy in an adaptive manner and to minimize side effects of chronic neurostimulation therapy. The results of the sensitivity analysis on stimulation parameters is a necessary step to optimize the design of the closed-loop control methods previously proposed by our team [11–13]. The definition of a patient-specific controller will require the application of a training phase, which could be based on most influent parameters (*P*_pw_, *P*_cur_ and *P*_npulses_) and their main interactions.

Limitations of the paper are mainly related to the ranges defined for stimulation parameters. These ranges were chosen to be easily implementable into an implantable neurostimulation device, delivering synchronous VNS. They were defined in order to preserve limited energy consumption, while avoiding side effects. In particular, the delay values were limited to take into account the technical properties of our current prototype, and kept within a safe interval, smaller than the average QT interval of the sheep. Although the conclusions proposed in this work are only valid inside these predefined ranges, the proposed ranking for stimulation parameters and their interactions provide precious information for defining VNS parameter optimization strategies. Moreover, in this work we analyzed acute cardiac effects of synchronous VNS, regardless of the neural pathway that provoked them (direct efferent effects or centrally-mediated afferent effects). It is possible that some of the studied parameter configurations provoke different afferent or efferent effects. Further experimental work is currently directed towards the evaluation of the effect of the main synchronous VNS parameters on sheep with intact and transected vagus nerves.

## Conclusion

This paper presented a formal analysis of the acute cardiac response to variations of the main parameters of cardiac-synchronized VNS. To our knowledge, this is the first work dedicated to the joint, quantitative exploration of chronotropic, dromotropic and inotropic effects in this context. In order to ensure an optimal evaluation of the parameter space, the experimental design was based on a Latin Hypercube sampling method, combined to a Gaussian process regression. Global sensitivity methods were used to propose a rank between parameters associated with a quantified analysis of the variability induced by each stimulation parameter. The quantified evaluation of interactive effects involved in the acute cardiac response to VNS provides original results with respect to previous works. Although an important inter-individual variability was observed, the predominant influence of current amplitude, pulse width, number of pulses and their interactions were clearly highlighted in the analysis for all sheep. Interpulse period and delay appear as negligible parameters within the range of parameter values studied in this work. However these parameter show non-negligible interacting effects. The proposed ranking between parameters will be exploited in our future works concerning the development of an adaptive closed-loop neuromudulator controller, seeking to optimize the therapy while minimizing side effects.

## Supporting Information

S1 Fig Example surface responses of the chronotropic, dromotropic and inotropic effects to VNS (respectively the first, second and third rows), generated with a Gaussian process regression for sheep 2. (PDF)

S2 Fig Example surface responses of the chronotropic, dromotropic and inotropic effects to VNS (respectively the first, second and third rows), generated with a Gaussian process regression for sheep 3. (PDF)

S3 Fig Example surface responses of the chronotropic, dromotropic and inotropic effects to VNS (respectively the first, second and third rows), generated with a Gaussian process regression for sheep 4. (PDF)

S4 Fig Example surface responses of the chronotropic, dromotropic and inotropic effects to VNS (respectively the first, second and third rows), generated with a Gaussian process regression for sheep 5. (PDF)

S5 Fig Example surface responses of the chronotropic, dromotropic and inotropic effects to VNS (respectively the first, second and third rows), generated with a Gaussian process regression for sheep 6. (PDF)

S1 Dataset Datasets containing the observed cardiac acute response (chronotropic, dromotropic and inotropic) for all sheep and all the VNS configurations analyzed in this study. (ZIP)

## References

[1] Ben-Menachem E, Manon-Espaillat R, Ristanovic R, Wilder B, Stefan H, Mirza W, et al Vagus nerve stimulation for treatment of partial seizures: 1. A controlled study of effect on seizures. First International Vagus Nerve Stimulation Study Group. Epilepsia. 1994;35(3):616–26. 10.1111/j.1528-1157.1994.tb02482.x 8026408

[2] Rush A, George M, Sackeim H, Marangell L, Husain M, Giller C, et al Vagus nerve stimulation (VNS) for treatment-resistant depressions: a multicenter study. Biol Psychiatry. 2000;47(4):276–86. 10.1016/S0006-3223(99)00304-2 10686262

[3] Guiraud D, Andreu D, Bonnet S, Carrault G, Couderc P, HagÃ¨ge A, et al Vagus nerve stimulation: state of the art of stimulation and recording strategies to address autonomic function neuromodulation. Journal of Neural Engineering. 2016;13(4):041002 10.1088/1741-2560/13/4/041002 27351347

[4] Klein H, Ferrari G. Vagus nerve stimulation: A new approach to reduce heart failure. Cardiol J. 2010;17(6):638–44. 21154273

[5] De Ferrari G. Vagal stimulation in heart failure. J Cardiovasc Transl Res. 2014;7(3):310–20. 10.1007/s12265-014-9540-1 24500409

[6] Vanoli E, De Ferrari GM, Stramba-Badiale M, Hull SS, Foreman RD, Schwartz PJ. Vagal stimulation and prevention of sudden death in conscious dogs with a healed myocardial infarction. Circulation Research. 1991;68(5):1471–81. 10.1161/01.RES.68.5.1471 2019002

[7] Borovikova LV, Ivanova S, Zhang M, Yang H, Botchkina GI, Watkins LR, et al Vagus nerve stimulation attenuates the systemic inflammatory response to endotoxin. Nature. 2000;405(6785):458–62. 10.1038/35013070 10839541

[8] Schwartz PJ, De Ferrari GM. Vagal stimulation for heart failure: background and first in-man study. Heart Rhythm. 2009;6(11 Suppl):S76–81. 10.1016/j.hrthm.2009.08.012 19880077

[9] Zannad F, De Ferrari G, Tuinenburg A, Wright D, Brugada J, Butter C, et al Chronic vagal stimulation for the treatment of low ejection fraction heart failure: results of the NEural Cardiac TherApy foR Heart Failure (NECTAR-HF) randomized controlled trial. Eur Heart J. 2015;36(7):425–33. 10.1093/eurheartj/ehu345 25176942 PMC4328197

[10] Premchand R, Sharma K, Mittal S, Monteiro R, Dixit S, Libbus I, et al Extended Follow-Up of Patients with Heart Failure Receiving Autonomic Regulation Therapy in the ANTHEM-HF Study. J Card Fail. 2015; 10.1016/j.cardfail.2015.11.002 26576716

[11] Romero-Ugalde HM, Rolle VL, Bel A, Bonnet JL, Guiraud D, Mabo P, et al. On-off closed-loop control of vagus nerve stimulation for the adaptation of heart rate. In: Engineering in Medicine and Biology Society (EMBC), 2014 Annual International Conference of the IEEE; 2014. p. 6262–5. 10.1109/EMBC.2014.6945060 25571428

[12] Romero-Ugalde HM, Ojeda D, Le Rolle V, Rossel O, Bonnet JL, Karam N, et al. Model-based design of control modules for neuromodulation devices. In: Neural Engineering (NER), 2015 7th International IEEE/EMBS Conference on; 2015. p. 462–5.

[13] Romero-Ugalde HM, Ojeda D, Rolle VL, Andreu D, Guiraud D, Bonnet JL, et al Model-based design and experimental validation of control modules for neuromodulation devices. Biomedical Engineering, IEEE Transactions on. 2015; 10.1109/TBME.2015.2498878 26571507

[14] Kong SS, Liu JJ, Hwang TC, Yu XJ, Zhao M, Zhao M, et al Optimizing the Parameters of Vagus Nerve Stimulation by Uniform Design in Rats with Acute Myocardial Infarction. Plos One. 2012;7(11):e42799 10.1371/journal.pone.0042799 23189120 PMC3506552

[15] Buschman HP, Storm CJ, Duncker DJ, Verdouw PD, van der Aa HE, van der Kemp P. Heart Rate Control Via Vagus Nerve Stimulation. Neuromodulation: Technology at the Neural Interface. 2006;9(3):214–20. 10.1111/j.1525-1403.2006.00062.x 22151709

[16] Dong E, Reitz BA. Effect of Timing of Vagal Stimulation on Heart Rate in the Dog. Circulation research. 1970;27(5):635–46. 10.1161/01.RES.27.5.635 5486240

[17] Thompson GW, Levett JM, Miller SM, Hill MRS, Meffert WG, Kolata RJ, et al Bradycardia Induced by Intravascular Versus Direct Stimulation of the Vagus Nerve. The Annals of Thoracic Surgery. 1998;65(3):637–42. 10.1016/S0003-4975(97)01351-9 9527187

[18] Levy M, Zieske H. Effect of Enhanced Contractility on the Left Ventricular Response to Vagus Nerve Stimulation in Dogs. Circulation research. 1969;24(3):303–11. 10.1161/01.RES.24.3.303 5766513

[19] Martin P, Levy MN, Matsuda Y. Fade of cardiac responses during tonic vagal stimulation. American Journal of Physiology—Heart and Circulatory Physiology. 1982;243(2):H219–25. 7114232 10.1152/ajpheart.1982.243.2.H219

[20] Henning RJ, Cheng J, Levy MN. Vagal stimulation decreases rate of left ventricular relaxation. American Journal of Physiology—Heart and Circulatory Physiology. 1989;256(2):H428–33. 2916675 10.1152/ajpheart.1989.256.2.H428

[21] Wallick DW, Levy MN, Felder DS, Zieske H. Effects of repetitive bursts of vagal activity on atrioventricular junctional rate in dogs. American Journal of Physiology—Heart and Circulatory Physiology. 1979;237(3):H275–81. 224714 10.1152/ajpheart.1979.237.3.H275

[22] Yang T, Jacobstein MD, Levy MN. Synchronization of automatic cells in S-A node during vagal stimulation in dogs. American Journal of Physiology—Heart and Circulatory Physiology. 1984;246(4):H585–91. 6720914 10.1152/ajpheart.1984.246.4.H585

[23] Yang T, Cheng J, Levy MN. Effects of the spatial dispersion of acetylcholine release on the chronotropic responses to vagal stimulation in dogs. Circulation research. 1990;67:844–51. 10.1161/01.RES.67.4.844 2208610

[24] Rousselet L, Rolle VL, Ojeda D, Guiraud D, Hagege A, Bel A, et al. Influence of Vagus Nerve Stimulation parameters on chronotropism and inotropism in heart failure. In: Engineering in Medicine and Biology Society (EMBC), 2014 36th Annual International Conference of the IEEE; 2014. p. 526–529. 10.1109/EMBC.2014.6943644 25570012

[25] Yoo P, Liu H, Hincapie J, Ruble S, Hamann J, Grill W. Modulation of heart rate by temporally patterned vagus nerve stimulation in the anesthetized dog. Physiological Reports. 2016;4(2):e12689 10.14814/phy2.12689 26811057 PMC4760392

[26] Yamakawa K, Rajendran PS, Takamiya T, Yagishita D, So EL, Mahajan A, et al Vagal nerve stimulation activates vagal afferent fibers that reduce cardiac efferent parasympathetic effects. Am J Physiol Heart Circ Physiol. 2015;309(9):H1579–90. 10.1152/ajpheart.00558.2015 26371172 PMC4666973

[27] Ardell JL, Rajendran PS, Nier HA, KenKnight BH, Armour JA. Central-peripheral neural network interactions evoked by vagus nerve stimulation: functional consequences on control of cardiac function. Am J Physiol Heart Circ Physiol. 2015;309(10):H1740–52. 10.1152/ajpheart.00557.2015 26371171 PMC4666982

[28] Andreu D, Guiraud D, Souquet G. A Distributed Architecture for Activating the Peripheral Nervous System. Journal of Neural Engineering. 2009;6 10.1088/1741-2560/6/2/026001 19213992

[29] Stocki R. A method to improve design reliability using optimal latin hypercube sampling. Computer Assisted Mechanics and Engineering Sciences. 2005;12(4):87–105.

[30] Dumont J, Hernández AI, Carrault G. Improving ECG beats delineation with an evolutionary optimization process. IEEE Trans Biomed Eng. 2010;57(3):607–15. 10.1109/TBME.2008.2002157 19171513

[31] Hernández AI, Carrault G, Mora F. Improvement of a P-wave detector by a bivariate classification stage. Transactions of the institute of measurement and control. 2000;22(3):231–42. 10.1177/014233120002200303

[32] Kleijnen J. Kriging metamodeling in simulation: A review. European Journal of Operational Research. 2009;192(3):707–16. 10.1016/j.ejor.2007.10.013

[33] Rasmussen CE, Williams CK. Gaussian processes for machine learning. the MIT Press; 2006.

[34] Roustant O, Ginsbourger D, Deville Y. DiceKriging, DiceOptim: Two R Packages for the Analysis of Computer Experiments by Kriging-Based Metamodeling and Optimization. Journal of Statistical Software. 2012;51(1):1–55. 10.18637/jss.v051.i0123504300

[35] Sobol I, Tarantola S, Gatelli D, Kucherenko S, Mauntz W. Estimating the approximation error when fixing unessential factors in global sensitivity analysis. Reliability engineering & systems safety. 2007;92(7):957–60. 10.1016/j.ress.2006.07.001

[36] Saltelli A, Ratto M, Andres T, Campolongo F, Cariboni J, Gatelli D, et al Global sensitivity analysis: the primer. John Wiley & Sons; 2008.

[37] Pujol G, Iooss B, with contributions from Khalid Boumhaout AJ, Veiga SD, Fruth J, Gilquin L, et al.. sensitivity: Global Sensitivity Analysis of Model Outputs; 2016. Available from: http://CRAN.R-project.org/package=sensitivity

[38] Chen S, Chiang C, Tai C, Wen Z, Lee S, Chiou C, et al Intracardiac stimulation of human parasympathetic nerve fibers induces negative dromotropic effects: implication with the lesions of radiofrequency catheter ablation. J Cardiovasc Electrophysiol. 1998;9(3):245–52. 10.1111/j.1540-8167.1998.tb00909.x 9554729

[39] Castoro M, Yoo P, Hincapie J, Hamann J, Ruble S, Wolf P, et al Excitation properties of the right cervical vagus nerve in adult dogs. Experimental Neurology. 2011;227(1):62–8. 10.1016/j.expneurol.2010.09.011 20851118

