# Supplementary material for: Sensitivity Analysis of Vagus Nerve Stimulation Parameters on Acute Cardiac Autonomic Responses: Chronotropic, Inotropic and Dromotropic Effects
Source: PLoS One. 2016 Sep 30;11(9):e0163734. doi: 10.1371/journal.pone.0163734 (PMC5045213; doi:10.1371/journal.pone.0163734)
Supplement: S5 Fig — (PDF) [file pone.0163734.s005.pdf]

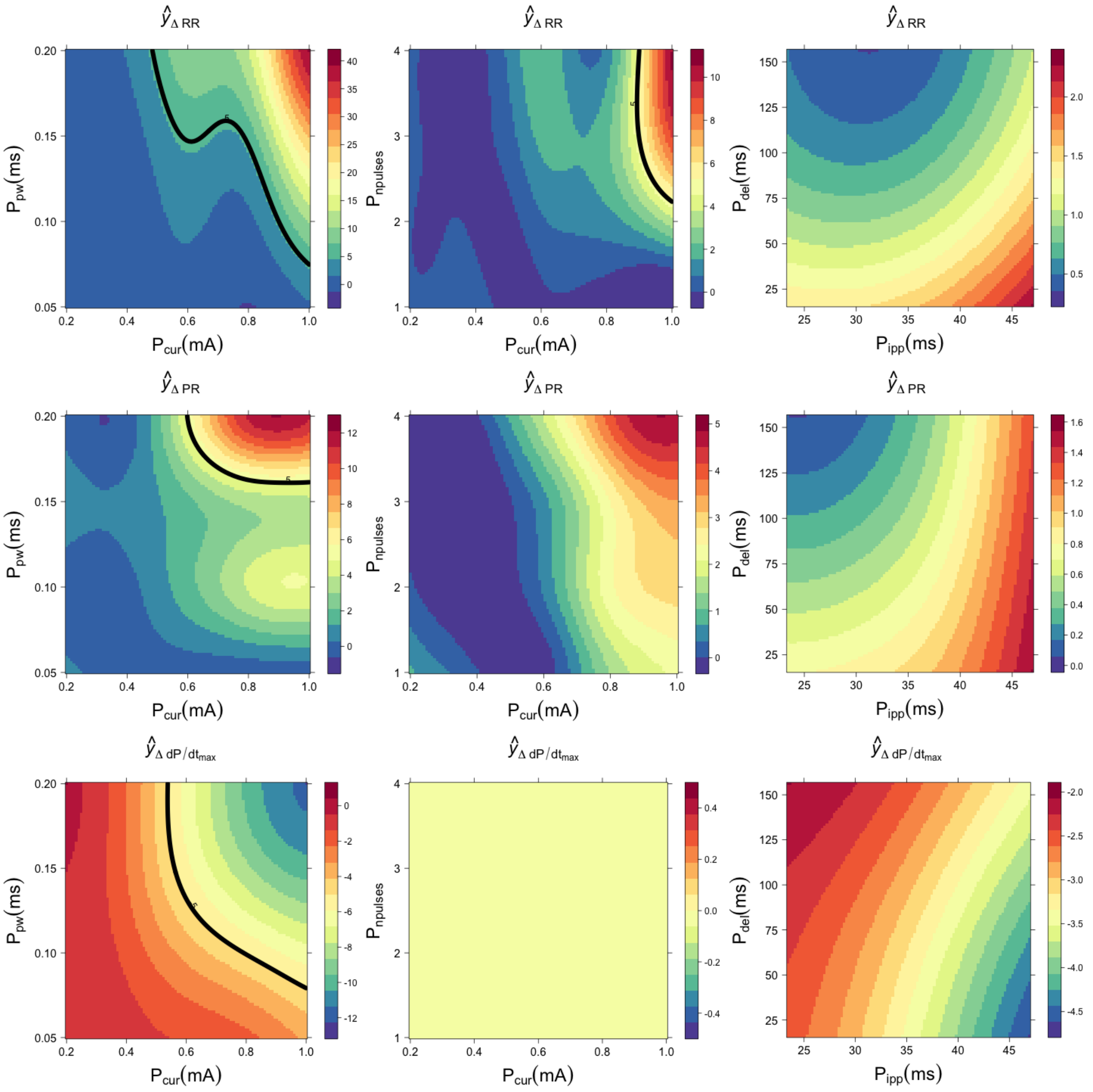

S5 Fig. Example surface responses of the chronotropic, dromotropic and inotropic effects to VNS (respectively the first, second and third rows), generated with a Gaussian process regression for sheep 6
